# Electronic diffusion in a normal state of high-Tc cuprate YBa_2_Cu_3_O_6+x_

**DOI:** 10.1073/pnas.2322670121

**Published:** 2024-03-12

**Authors:** Jure Kokalj

**Affiliations:** ^a^Department of Theoretical Physics, Jožef Stefan Institute, Ljubljana 1000, Slovenia; ^b^Faculty of Civil and Geodetic Engineering, Department of Mathematics and Physics, University of Ljubljana, Ljubljana 1000, Slovenia

**Keywords:** superconducting cuprates, electronic transport, diffusion, heat conductivity

## Abstract

The bad metallic phase with resistivity above the Mott–Ioffe–Regel (MIR) limit, which appears also in cuprate superconductors, was recently understood by cold atom and computer simulations of the Hubbard model via charge susceptibility and charge diffusion constant. However, since reliable simulations can be typically done only at temperatures above the experimental temperatures, the question for cuprate superconductors is still open. This paper addresses this question by resorting to heat transport, which allows for the estimate of electronic diffusion and it further combines it with the resistivity to estimate the charge susceptibility. The doping and temperature dependencies of diffusion constant and charge susceptibilities are shown and discussed for two samples of YBa_2_Cu_3_O_6+x_. Results indicate strongly incoherent transport, mean free path corresponding to the MIR limit for the underdoped sample at temperatures above ~200 K and significant effect of the charge susceptibility on the resistivity.

Understanding the resistivity in the normal state of high-Tc cuprates poses one of the central challenges in strongly correlated systems for almost four decades and could be a crucial step for understanding the superconductivity. Resistivity shows unusually high values reaching well above the proposed Mott–Ioffe–Regel (MIR) limit at high temperatures (e.g., above 300 K for low hole dopings in YBa_2_Cu_3_O_6+x_). Such bad-metallic behavior was recently understood with cold atoms (1) and computer (2, 3, 4) simulations of Hubbard model in terms of strongly suppressed charge susceptibility $\chi_{c}$, which results in large resistivity *ρ* via the Nernst–Einstein relation $\rho =1/(D_{c}\chi_{c})$. It was further shown that besides the charge diffusion constant $D_{c}$, which decreases with *T* and saturates at very high *T*, also $\chi_{c}$ decreases with *T* and importantly affects the *T* dependence of *ρ*. Model simulations correspond to very high *T* and merely touch cuprate’s experimental high-*T* regime. This raises an important question. Is the behavior observed in simulations for high *T*, e.g., the importance of the *T*-dependence of $\chi_{c}$ stretched into the experimental lower *T* regime for cuprates? This seems plausible, since for some dopings *ρ* shows no qualitative change from the highest *T* all the way down to the superconducting transition temperature *T_c_*. This question could be answered with data either on $\chi_{c}$ or $D_{c}$, but both are still elusive for experimental probing.

Here, we resort to another line of research with many remarkable discoveries for cuprates: The study of thermal properties. Measured specific heat c and its electronic part $c_{\mathrm{el}}$ stay robust with practically no corrections since the early measurements (5). Heat conductivity κ offered an early support for the d-wave pairing in cuprates (6) and revealed increased heat conduction below *T_c_* triggering hot debate (7, 8) on its origin: increased mean-free-path of either phonons or non-superconducting electrons. The latter was strongly supported by the increased infrared conductivity (9, 10). For the insulating parent compound, the magnonic κ was determined as the difference between the measured in-plane and out-of-plane conductivities allowing the extraction (11) of very long magnonic mean free paths (longer than 100 unit cells). Introductions of impurities (10, 11), defects (12), and magnetic fields (11) were used to reveal the conduction and scattering mechanisms. Furthermore, directly measured heat diffusion constant was discussed in terms of Planckian dissipation and a soup-like mixture of electrons and phonons (13).

## Results and Discussion

**Fig. 1.**
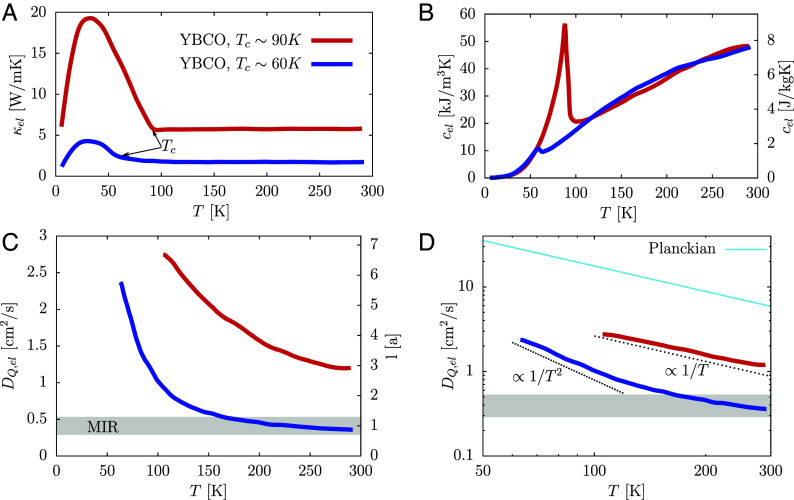
(*A*) Electronic heat conductivity $\kappa_{\mathrm{el}}$ for two YBCO samples ($T_{c}∼60\;K$ and $T_{c}∼90\;K$) from ref. 10. Transition temperatures $T_{c}$ are indicated. (*B*) Electronic specific heat as measured for similar samples from ref. 5. (*C*) Calculated electronic heat diffusion constant $D_{Q,el}$ for two samples showing decrease with *T* and for $T_{c}∼60\;K$ sample also values and tendency for saturation close to the MIR limit at higher *T*. Due to some uncertainty in definition of MIR limit, it is shown with 30% range from l=a. Right *y*-axis shows estimated mean free path. (*D*) $D_{Q,el}$ in logarithmic scale compared with $1/T^{2}$, 1/T and suggested Planckian lower bound for a prefactor α=1.

Heat transport is special in a sense that all quantities in its Nernst–Einstein equation $\kappa =D_{Q}c$ can be independently measured—even the heat diffusion constant $D_{Q}$ (13). The challenge however is the separation of phononic $\kappa_{\mathrm{ph}}$ and electronic $\kappa_{\mathrm{el}}$ contributions to the total conductivity $\kappa =\kappa_{\mathrm{ph}}+\kappa_{\mathrm{el}}$. Takenaka et al. (10) carefully analyzed the O and Zn doping dependence of κ for YBa_2_Cu_3_O_6+x_ (YBCO) and obtained good estimates for $\kappa_{\mathrm{el}}$ (Fig. 1*A*). We use these data, together with the data for $c_{\mathrm{el}}$ (Fig. 1*B*) from Loram et al. (5) to estimate the electronic heat diffusion constant $D_{Q,el}$ via the Nernst–Einstein relation $D_{Q,el}=\kappa_{\mathrm{el}}{/c}_{\mathrm{el}}$. The resulting $D_{Q,el}$ for two measured compounds with $T_{c}∼60\;K$ (x ∼ 0.68) and $T_{c}∼90\;K$ (x ∼ 0.93) in the non-superconducting regime is shown in Fig. 1*C*. $D_{Q,el}$ decreases with increasing *T* as expected, since it is related to the mean free path l via $D_{Q,el}=vl/2$ and l normally decreases with increasing *T* due to increasing scattering. Here, v is a mean quasiparticle velocity. By using its estimate $v=2.15\times 10^{5}$ m/s (13) and setting l to minimal value (l∼a with a being a lattice constant) one obtains a MIR limit for $D_{Q,el}$ (Fig. 1*C*). Remarkably, $D_{Q,el}$ for the $T_{c}∼60\;K$ compound decreases to such value already at relatively low T∼200 K and shows signs of saturation close to MIR value at higher T. This reveals strongly incoherent behavior in such regime. From value of $D_{Q,el}$ and v one can estimate l (right y axis in Fig. 1*C*). This average value of l is small and reaches l∼6a at lowest $T∼T_{c}$. T dependence of $D_{Q,el}$ can be related to the T dependence of scattering time τ via $D_{Q,el}=v^{2}\tau /2$. In Fig. 1*D* we show that for $T_{c}∼60\;K$ compound the behavior at lowest T is most consistent with Fermi liquid like scattering $1/\tau \propto T^{2}$, advocated, e.g., in ref. 14, while the $T_{c}∼90\;K$ compound shows more 1/τ∝T like behavior. We show in Fig. 1*D* also the comparison to the suggested (15) lower bound on diffusion $D_{p.b.}=\alpha \;\hbar \;v^{2}/\left(2k_{B}T\right)$ obtained from a Planckian timescale $\tau =\alpha \;\hbar \;/(k_{B}T)$. $D_{p.b.}$ is shown for a numerical prefactor α of the order unity set to α=1. $D_{Q,el}$ is much smaller and the consistency with $D_{p.b.}$ for a $T_{c}∼90\;K$ compound requires α∼0.2, while smaller α∼0.05 is needed for consistency with $T_{c}∼60\;K$ compound.

**Fig. 2.**
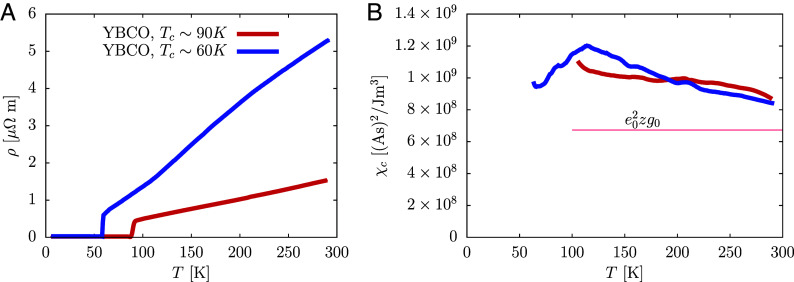
(*A*) Resistivity ρ for the same YBCO samples as in Fig. 1. (*B*) Via the Nernst–Einstein relation deduced charge susceptibility $\chi_{c}$ and its comparison with theoretical estimate. $\chi_{c}$ shows notable *T* and doping dependence, as well as cusp at presumably CDW transition.

By having values for $D_{Q,el}$, we can make a step toward estimating $\chi_{c}$. $D_{c}$ and $D_{Q,el}$ typically do not behave the same, but they are related. It was found that within a dynamical mean field theory (16), they are at low T related by $D_{c}={\mathrm{fD}}_{Q,el}$, with a factor $f=1/z^{2}$ and z being a quasiparticle weight. By assuming a constant factor f in regime of measured data and setting it to an approximate value $f=\frac{1}{0.4^{2}}$, we obtain a sensible approximation for $D_{c}$. This can be used together with the data on resistivity (10) (Fig. 2*A*) to calculate $\chi_{c}$ via the Nernst-Einstein relation $\chi_{c}=1/\left(\rho D_{c}\right)$. The resulting $\chi_{c}$ is shown in Fig. 2*B*. It is compared to the theoretical estimate $\chi_{c}\approx e_{0}^{2}zg_{0}$, with $g_{0}$ being a non-interacting density of states. $\chi_{c}$ is larger than theoretical estimate and in addition shows some *T* dependence. A clear cusp is seen for $T_{c}∼60\;K$ compound at T∼120 K presumably corresponding to the charge density-wave (CDW) phase transition (17). More relevant for our discussion is its *T* and doping dependence above such *T*. While both compounds show decreasing $\chi_{c}$ with increasing *T*, the decrease for $T_{c}∼60\;K$ compound is stronger. From 200 K to 300 K, its $\chi_{c}$ decreases by about 15%, which is comparable to the decrease of $D_{c}$ for about 20% (together they give an increase of ρ for about 50%). This is similar to the behavior observed in the Hubbard model (1). *T* dependence of $\chi_{c}$ therefore plays an important role for ρ in YBCO and may turn out to be even greater at higher *T* as $D_{c}$ is expected to be saturating while ρ increases further. This agrees well with the picture from the optical conductivity (18), in which the increase of resistivity with increasing *T* in the bad metallic phase is due to the transfer of low frequency spectral weight to higher frequencies, while the mean free path is saturating. It is interesting to note, that with decreasing doping, $\chi_{c}(T∼150\;K)$ is increasing, which is consistent with the phase separation tendency, for which $\chi_{c}$ diverges at some point in the extended phase diagram (19). We also note that the Nernst–Einstein relation is applicable also to the pseudogap phase as it relies (2, 3, 15) only on the normal behavior with some scattering or current relaxation mechanism leading to the finite diffusion constant.

One should keep in mind that the analysis here is based on experimental data, which have some degree of uncertainty and that the properties are averaged over all carriers with no differentiation, e.g., nodal vs. anti-nodal Fermi surface parts. However, the main outcomes like strongly incoherent electronic transport close to expected MIR saturation and the first estimate of $\chi_{c}$ suggesting notable *T* and doping dependence could withstand future tests. The presented behavior and insight also hint on possible better understanding and calls for further experimental efforts to pin down at least one additional quantity, either charge diffusion constant $D_{c}$ or charge susceptibility $\chi_{c}$.

## Materials and Methods

Some details on calculations can be found in *SI Appendix*.

All the data are taken from references and results calculated as explained in the text.

## Supplementary Material

Appendix 01 (PDF)

## Data Availability

Previously published data were used for this work (5, 10). All other data are included in the manuscript and/or *SI Appendix*.
