# Supplementary material for: Electronic diffusion in a normal state of high-Tc cuprate YBa2Cu3O6+x
Source: Proc Natl Acad Sci U S A. 2024 Mar 12;121(12):e2322670121. doi: 10.1073/pnas.2322670121 (PMC10963013; doi:10.1073/pnas.2322670121)
Supplement: Supplementary file 1 — Appendix 01 (PDF) [file pnas.2322670121.sapp.pdf]

Supplementary Information:

Extended materials and methods

$\chi_c = e_0^2 dn/d\mu$ with n being a density of electrons and μ the chemical potential. χ_c can be for non-interacting particles related to the density of states at the Fermi energy, while it is renormalized in the presence of interactions (1,2). First approximation gives $\chi_c \approx e_0^2 z g_0$ and we estimate the non-interacting density of states as $g_0 = \sqrt{\frac{2}{\pi}} \frac{2}{\hbar c a v_0}$ corresponding to an isotropic 2D Fermi surface. We use bare band velocity $v_0 = 5.2 \times 10^5$ m/s estimated from LDA band dispersions (3), lattice constants $c = 11.68$ Å and $a = 3.82$ Å (4), half-filled band and two Cu-O planes within c. For unit cell volume the lattice constant $b = 3.89$ Å (4) is also used and mass density 6.3 g/cm^3 for transformation of units between left and right *y* axes in Fig. 1B.

References

1. J. Kokalj, Ross H. McKenzie, Thermodynamics of a Bad Metal–Mott Insulator Transition in the Presence of Frustration. Phys. Rev. Lett **110**, 206402 (2013).
2. M. Ulaga *et al.*, Thermal conductivity and heat diffusion in the two-dimensional Hubbard model. Phys. Rev. B **106**, 245123 (2022).
3. I. S. Elfimov *et al.*, Theory of Fermi-surface pockets and correlation effects in underdoped YBa2Cu3O6.5. Phys. Rev. B **77**, 060504(R) (2008).
4. S.I. Bondarenko *et al.*, High-temperature superconductors of the family (RE)Ba2Cu3O7-d and their application (Review Article). Fiz. Nizk. Temp. 43, 1411 (2017).
